# An updated algorithm for an effective choice of continuous glucose monitoring for people with insulin-treated diabetes

**DOI:** 10.1007/s12020-023-03473-w

**Published:** 2023-09-07

**Authors:** Maria Ida Maiorino, Raffaella Buzzetti, Concetta Irace, Luigi Laviola, Nicola Napoli, Dario Pitocco, Katherine Esposito, on behalf of LIVE CGM working group

**Affiliations:** 1 Unit of Endocrinology and Metabolic Diseases, University Hospital Luigi Vanvitelli, Piazza Miraglia 2, 80138 Naples, Italy; 2 Department of Advanced Medical and Surgical Sciences, University of Campania Luigi Vanvitelli, Piazza Miraglia 2, 80138 Naples, Naples, Italy; 3 Department of Experimental Medicine, “Sapienza” University of Rome, Viale Regina Elena 324, 00161 Rome, Italy; 4 Department of Health Science, University Magna Graecia, Viale Europa, 88100 Catanzaro, Italy; 5 Department of Precision and Regenerative Medicine and Ionian Area, Section of Internal Medicine, Endocrinology, Andrology and Metabolic Diseases, University of Bari Aldo Moro, Piazza Giulio Cesare 11, 70124 Bari, Italy; 6 Fondazione Policlinico Universitario Campus Bio-Medico, Research Unit of Endocrinology and Diabetes, Department of Medicine and Surgery, Università Campus Bio-Medico of Rome, Via Alvaro del Portillo 21, 00128 Rome, Italy; 7 Diabetes Care Unit, Department of Translational Medicine and Surgery, Università Cattolica del Sacro Cuore, Fondazione Policlinico Universitario Agostino Gemelli IRCCS, Largo Francesco Vito 1, 00168 Rome, Italy

**Keywords:** Continuous glucose monitoring, rtCGM, isCGM, Algorithm, Insulin-treated diabetes, Hypoglycemia

## Abstract

**Purpose:**

Continuous Glucose Monitoring (CGM) is a key tool for insulin-treated people with diabetes (PwD). CGM devices include both real-time CGM (rtCGM) and intermittently scanned CGM (isCGM), which are associated with an improvement of glucose control and less hypoglycemia in clinical trials of people with type 1 and type 2 diabetes.

**Methods:**

This is an expert position to update a previous algorithm on the most suitable choice of CGM for insulin-treated PwD in light of the recent evidence and clinical practice.

**Results:**

We identified six different clinical scenarios, including type 1 diabetes, type 2 diabetes, pregnancy on intensive insulin therapy, regular physical exercise, new onset of diabetes, and frailty. The use of rtCGM or isCGM is suggested, on the basis of the predominant clinical issue, as suboptimal glucose control or disabling hypoglycemia, regardless of baseline HbA_1c_ or individualized HbA_1c_ target.

**Conclusion:**

The present algorithm may help to select the best CGM device based on patients’ clinical characteristics, needs and clinical context, offering a further opportunity of a “tailored” therapy for people with insulin-treated diabetes.

## Introduction

Continuous Glucose Monitoring (CGM) is a key tool for insulin-treated people with diabetes (PwD) [1, 2] measuring interstitial glucose level on a near-continuous basis, and providing information about glucose trend and rate of change. When appropriately used, CGM helps PwD make therapeutic decision before meals, exercise and any other condition that may influence glucose levels. A recent meta-analysis demonstrated that PwD, both type 1 and type 2, using CGM systems experience fewer hyperglycemic and hypoglycemic episodes, with a modest but significant decrease in glycated hemoglobin (HbA_1C_) levels [3].

A typical CGM requires three elements, the sensor, the transmitter, and the receiver. The sensor is placed under the skin and sends glucose readings to the transmitter attached to the skin above the sensor. The transmitter wirelessly sends glucose measurements to the receiver, which is a dedicated device or smart device (smartphone and smartwatch) by an App. Current available CGM devices include both real-time CGM (rtCGM), which continuously provides current and predicted glucose levels, and intermittently scanned CGM (isCGM), which displays glucose values when the transmitter is swiped by a reader or a smartphone. Moreover, a third type of CGM, the professional or “blinded” CGM is used by health care professionals as a diagnostic tool to retrospectively evaluate glycemic patterns and trends. CGM systems have distinctive characteristics, sensor lifetime (from 7 days to 6 months), accuracy, smartphone compatibility, insulin pump integration, costs, alarms and alerts, and user-friendliness. Furthermore, the systems may require different calibration conditions, have an adjunctive or non-adjunctive use, and can be integrated with insulin pump. Finally, the glucose measurements are processed by different software which summarize and display the data according to the international consensus on time in range and AGP (Ambulatory Glucose Profile) consensus [4]. (Table 1). The choice of a CGM system will depend on patient needs and preferences, system features, patient characteristics including the type of diabetes, individual skills, glucose control, concomitant morbidities, frailty, pregnancy, and cost.

**Table 1:** Characteristics of currently available CGM devices

CGM type/sensor	Reading period/Sensor lifetime/Transmitter lifetime	Calibrations (n/day)	Smartphone compatibility/ CSII integrated (SAP/AP)	MARD	Insertion site	% 15/15 mg/dL overall^e^	% 20/20 mg/dL overall^f^	Data management	Adjunctive use/Non adjunctive use	Use in pregnancy^i^	Intended populations
rtCGM/Dexcom G6	10 days/10 days/ 3 months	0	yes/yes	9%	Abdomen, arm, upper gluteus^d^	83.3%	93.9%	Clarity/Diasend/ Glooko	Non adjunctive use	Indicated	2 years and older
rtCGM/Dexcom G7	10 days/10 days^b^ (+12 h tolerance)	0	yes/yes	8.2%	Abdomen, arm, upper gluteus^d^	89.6%^g^	95.3%^g^	Clarity/ Glooko	Non adjunctive use	Indicated	2 years and older
rtCGM/TouchCare A7+	10 days /10 days/ 12 months	1	yes/yes	9%	Abdomen, upper gluteus	NA	NA	EasyView Pro	Non adjunctive use	Not specified	2 years and older
rtCGM/Eversense E3^a^	180 days/ 180 days/12 months	2/1^c^	yes/no	8.5%	Upper arm	85.6%	92.9%	Eversense DMS	Non adjunctive use	Not indicated	18 years and older
rtCGM/GlucoMen Day	14 days/14 days/ 5 years	1	yes/no	9.6%	Abdomen	NA	NA	Glucolog/Diasend/ Glooko	Non adjunctive use	Indicated	6 years and older
rtCGM/Glunovo i3/Flash	14 days/14 days/ 3 years	0	yes/no	10.3%	Abdomen	79.3%	89.7%	IrisHealthCare	Adjunctive use	Indicated	2 years and older
rtCGM/Guardian sensor 3	7 days/7 days/ 12 months	2	no/yes	9.1%	Abdomen, arm	78.8%	88.2%	Carelink	Adjunctive use^h^	Not specified	2 years and older
rtCGM/Guardian sensor 4	7 days/7 days/ 12 months	0	no/yes	10.6%	Abdomen, arm, upper gluteus^d^	NA	NA	Carelink	Non adjunctive use	Not specified	2 years and older
isCGM/Freestyle libre	14 days/14 days^b^	0	yes/no	9.2%	arm	NA	NA	Libreview	Non adjunctive use	Indicated	4 and older
isCGM/Freestyle libre 2	14 days/14 days^b^	0	yes/no	9.2%	arm	86.3%	93.2%	Libreview	Non adjunctive use	Indicated	4 and older
rtCGM/Freestyle libre 3	14 days/14 days^b^	0	yes/no	7.9%	arm	89.1%	94.7%	Libreview	Non adjunctive use	Indicated	4 and older

^a^Implantable sensor

^b^The sensor and the transmitter work as one unit

^c^Eversense E3 requires 2 calibration/day for the first 21 days of use and after 1 calibration /day

^d^Upper gluteus refers to pediatric use between 2 and 17 years of age

^e^Overall agreement rate between paired sensor–reference values within ±15%/15%

^f^Overall agreement rate between paired sensor–reference values within ±20%/20%

^g^Overall agreement rate for arm-placed sensors

^h^Approved for non adjunctive use only in conjunction with MiniMed 780 G

^i^use in pregnancy according to the technical sheet. *AP* Artificial pancreas, *CGM* Continuous glucose monitoring, *CSII* Continuous subcutaneous insulin infusion, *isCGM* Intermittently scanned glucose monitoring, *MARD* Mean absolute relative difference, *rtCGM* Real-time continuous glucose monitoring, *SAP* Sensor augmented pump

CGM is strongly recommended for PwD treated with intensive insulin therapy. Both international [2, 5] and Italian [6] guidelines highlight the importance of selecting and educating patients and caregivers to the use of CGM. The guidelines identify poor glucose control and hypoglycemia as the main indications for CGM use, especially in case of problematic hypoglycemia (frequent, severe, nocturnal, not perceived).

We aimed our current expert position to update the prior published algorithm on the most suitable choice of CGM for insulin-treated PwD in light of the recent evidence reporting the efficacy and safety of CGM in type 1 and type 2 diabetes [7–9], and clinical practice.

## The updated algorithm

The previous algorithm [10] has been extensively revised in order to support the use of rtCGM and isCGM in different clinical scenarios. Our suggestions based on evidence and good clinical practice will allow a more comprehensive approach, tailoring possible CGM solutions according to the clinical issues and needs of PwD.

The structured self-monitoring blood glucose (SMBG) (at least four fingerpicks per day) together with reinforced patient-centered education remains the first-line glucose monitoring strategy for adults with diabetes on intensive insulin therapy [2].

The second-line strategy with rtCGM or isCGM is suggested, regardless of baseline HbA_1c_ or individualized HbA_1c_ target, on the basis of the predominant clinical issue, as suboptimal glucose control or disabling hypoglycemia.

Six different clinical scenarios have been identified: type 1 diabetes, type 2 diabetes, pregnancy on intensive insulin therapy, regular physical exercise, new onset of diabetes, and frailty. The choice of CGM system is based on two main factors, the ‘suboptimal glucose control’ and risk of ‘disabling hypoglycemia’. The suboptimal glucose control is referred to HbA_1c_ not at desired target, and disabling hypoglycemia to history of frequent severe hypoglycemia, nocturnal hypoglycemia, and hypoglycemia unawareness. Additional CGM features are also taken into account for each scenario, as reported below.

### First scenario: type 1 diabetes

**Fig. 1:**
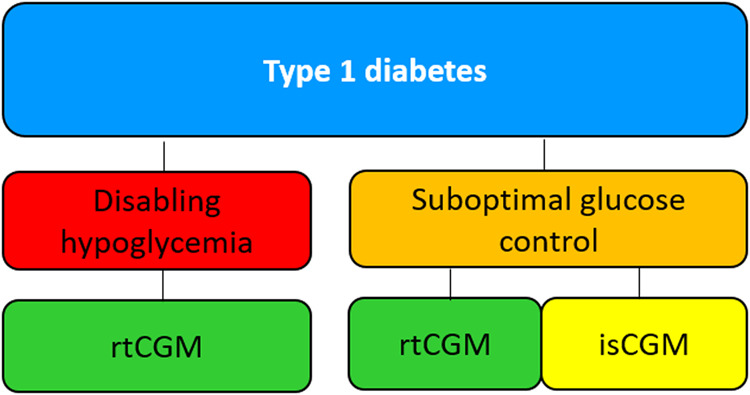
Suggested approach for the choice of CGM in type 1 diabetes

Figure 1. In people with type 1 diabetes with disabling hypoglycemia, or previous episodes of diabetic ketoacidosis (DKA), we suggest the rtCGM to customize alerts and alarms in order to predict impending hypoglycemia, acting quickly and avoid a potentially serious event. Systems with high accuracy in the low glucose range are recommended (Table 3). In the case of suboptimal glucose control, isCGM and rtCGM can both be suggested, depending on individual skills, patient preferences, smart devices availability, and costs.

CGM represents a fundamental therapeutic tool in type 1 diabetes since it allows to address the potential issues related to the "timing" of insulin administered at meals, the insulin-carbohydrate ratio (and more generally, the influence of other nutrients including proteins and fats) and the management of night period. In RCTs of adults with type 1 diabetes, both isCGM and rtCGM are effective in increasing the TIR and reducing the time spent in hypoglycemia (time below range, TBR) [7, 8, 11–13]. Clinical benefits in terms of improved glucose control have been reported for youth and middle aged adults with poor diabetes control (HbA_1c_ ranging between 7.5%–11.0%) using rtCGM, and middle aged adults with HbA_1c_ closer to optimal range (6.8–8.7%) using isCGM (Table 2). Specifically, rtCGM with predictive alerts and alarms was associated with a reduction of a number of hypoglycemic events [14], including severe hypoglycemia [15, 16], decrease in TBR [15–19], and an improvement in hypoglycemia awareness [15, 16, 20] in middle aged adults with type 1 diabetes prone to hypoglycemia or impaired awareness of hypoglycemia, and HbA_1c_ ranging between 7.5% and 8.2% (Table 2). Since hypoglycemia is the limiting factor of diabetes therapy and a major concerns for people with diabetes and their families, reducing both level 1 and level 2 hypoglycemia is critical to improve glucose daily oscillations, quality of life and diabetes distress and prevent serious hypoglycemic episodes occurring with loss of consciousness or seizures, respectively. Moreover, clinicians should be aware of the accuracy of the used device, in order to choice the systems with high accuracy in presence of persistent of prolonged hypoglycemia (Table 3).

**Table 2:** Suggested scenarios for using rtCGM or isCGM

Suggested scenario for using rtCGM	Demographic/clinical features	Suggested scenario for using isCGM	Demographic/clinical features
• Type 1 diabetes with disabling hypoglycemia ± suboptimal glucose control	• Adolescents, young adults, middle aged adults, HbA_1c_ 7.5–9.0%	• Type 1 diabetes with suboptimal glucose control	• Middle aged adults, HbA_1c_ 6.8–8.7%
• Type 2 diabetes with disabling hypoglycemia ± suboptimal glucose control	• Adults aged 35–79 years with most benefits in those ≥ 65 years, HbA_1c_ 7.8–11–0%	• Diabetes onset	• Youth with type 1 or type 2 diabetes, adults with type 2 diabetes
• Diabetes and pregnancy on intensive insulin therapy with disabling hypoglycemia and/or suboptimal glucose control	• Pregnant or planning pregnancy women, women with gestational diabetes	• Type 2 diabetes with suboptimal glucose control	• Adults, HbA_1c_ 7.5–12.0%
• Regular physical exercise with disabling hypoglycemia ± suboptimal glucose control	• Young adults with type 1 diabetes	• Diabetes and pregnancy on intensive insulin therapy with suboptimal glucose control	• Women with gestational diabetes
• Frail people with diabetes and disabling/unawareness hypoglycemia ± suboptimal glucose control according to wearability	• Older adults (≥ 65 years), people with CKD or cystic fibrosis	• Regular physical exercise with suboptimal glucose control^a^	• Adults with type 2 diabetes
		• Frail people with diabetes and suboptimal glucose control according to wearability^b^	• Older adults with type 1 or type 2 diabetes

*rtCGM* Real-time continuous glucose monitoring, *isCGM* Intermittently scanned glucose monitoring

^a^For FreeStyle Libre 2, as for the presence of predictive alarms, the scenario includes regular physical exercise with suboptimal glucose control ± disabling hypoglycemia

^b^for FreeStyle Libre 2, as for the presence of predictive alarms, the scenario includes frail people with diabetes and awareness hypoglycemia ± suboptimal glucose control according to wearability

**Table 3:** Accuracy of CGM decvices across glucose range

Sensor type	Accuracy across glucose ranges (20%/20%)		Key-note aspects	References
Dexcom G6	< 70 mg/dL	90.8	Accuracy has been evaluated in adults with both type 1 and type 2 diabetes and in diabetic children and adolescents	Garg et al., Diabetes Technol Ther. 2022;24(6):373–380; Laffel LM et al., J Diabetes Sci Technol. 2022;19322968221091816; Wadwa RP et al., Diabetes Technol Ther. 2018;20(6):395–402; Welsh JB et al., J Diabetes Sci Technol. 2022;19322968221099879.
	70–180 mg/dL	94.3		
	181–250 mg/dL	92.9		
	> 250 mg/dL	96.2		
Dexcom G7	40–60 mg/dL	91.9		
	61–80 mg/dL	96.5		
	81–180 mg/dL	93.6		
	181–300 mg/dL	96.0		
	301–400 mg/dL	99.1		
TouchCare A6/7+	< 70 mg/dL	72.0	Accuracy has been evaluated in one study including adults with both type 1 and type 2 diabetes (sensor A6)	Zhou J et al., J Diabetes Investig. 2018;9(2):286–293.
	70–180 mg/dL	88.2		
	> 180 mg/dL	93.0		
Eversense E3	40–60 mg/dL	89.4	Accuracy has been evaluated in multinational, multicenter studies including adults with both type 1 and type 2 diabetes	Garg SK et al., Diabetes Technol Ther. 2022;24(2):84–92; Kropff J et al., Diabetes Care. 2017;40(1):63–68.
	61–80 mg/dL	92.2		
	81–180 mg/dL	90.9		
	181–300 mg/dL	94.7		
	301–350 mg/dL	96.5		
GlucoMen Day^a^	40–70 mg/dL	20.2	Accuracy has been evaluated in one study including few adults with type 1 diabetes	Hochfellner DA et al., Biosensors (Basel). 2022;12(2):106.
	71–99 mg/dL	15.2		
	100–200 mg/dL	13.4		
	201–400 mg/dL	12.2		
Glunovo i3/Flash	< 54 mg/dL	42.3	Accuracy has been evaluated in one study including adults with both type 1 and type 2 diabetes	Meng R et al., Diabetes Ther. 2021;12(12):3153–3165.
	54–70 mg/dL	66.6		
	70–180 mg/dL	89.0		
	180–250 mg/dL	92.4		
	> 250 mg/dL	91.6		
Guardian sensor 3/4	≤ 70 mg/dL	93.1	Accuracy has been evaluated in studies including few adolescents and adults with both type 1 and type 2 diabetes and in children with type 1 diabetes	Christiansen MP et al., Diabetes Technol Ther. 2017;19(8):446–456; Slover RH et al., Diabetes Technol Ther. 2018;20(9):576–584.
	70–180 mg/dL	91.3		
	> 180 mg/dL	92.9		
Freestyle libre^a^	< 72 mg/dL	20.3	Accuracy has been evaluated in studies including adults with both type 1 and type 2 diabetes, pregnant women with diabetes and children with type 1 diabetes	Bailey T et al., Diabetes Technol Ther. 2015;17(11):787–94; Olafsdottir AF et al., Diabetes Technol Ther 2017;19:164–172; Fokkert MJ et al., BMJ Open Diabetes Res Care 2017; 5:e000320; Edge J et al., Arch Dis Child. 2017;102:543–549; Scott E et al., Diabetes Techol Ther 2017;19:(Suppl. 1):A84– A84.
	72–180 mg/dL	14.7		
	> 180 mg/dL	9.6		
Freestyle libre 2	< 70 mg/dL	98.4	Accuracy has been evaluated in one study including adults with both type 1 and type 2 diabetes	Alva S et al., J Diabetes Sci Technol. 2022;16(1):70–77.
	70–180 mg/dL	86.8		
	> 180 mg/dL	95.0		
Freestyle libre 3	<54 mg/dL	80.0	Accuracy has been evaluated in one study including children, adolescents and adults with both type 1 and type 2 diabetes	Alva S et al., Diabetes Ther. 2023;14(4):767–776.
	54–69 mg/dL	95.2		
	70–180 mg/dL	91.5		
	181–250 mg/dL	96.8		
	250–350 mg/dL	100.0		
	> 350 mg/dL	100.0		

^a^Referred as MARD across glucose range in people with type 1 diabetes

### Second scenario: diabetes onset

**Fig. 2:**
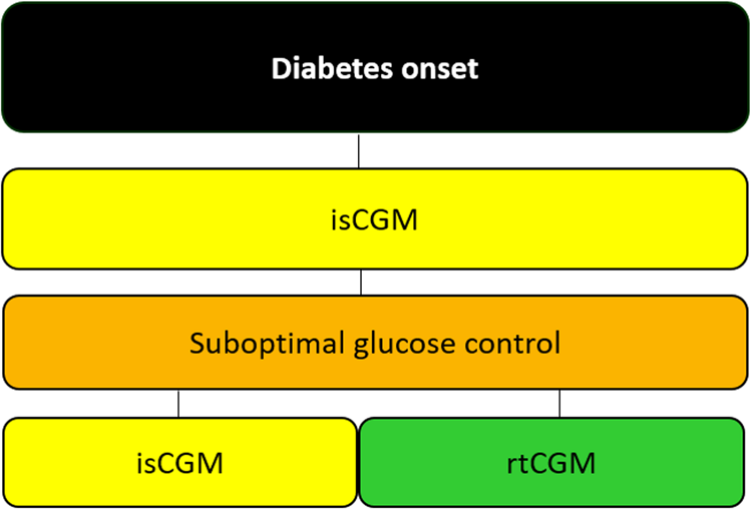
Suggested approach for the choice of CGM at diabetes onset

The isCGM should be proposed in each patient at the onset of diabetes when insulin therapy is required (Fig. 2). Its use should be also considered in people with a recent diagnosis of diabetes presenting prevalent hyperglycemia or in people with autoimmune diabetes in the stage of “honeymoon” with reduced insulin requirements. In the presence of frequent and severe hypoglycemia (with or without recognized precipitating cause) and poor glucose control despite intensive insulin therapy, rtCGM may be used due to the possibility of customizable alerts/alarms that allow patients to recognize in advance the effect of actions/factors that influence glucose variability. In people who are unwilling or unable to engage in the routine use of CGM, the occasional use of isCGM may be suggested to evaluate glucose trends and to guide the related therapeutic choices.

Preventing diabetic complications and optimizing the quality of life are both goals of diabetes therapy. A person-centered approach, tailored on both individual preferences and needs, is essential for effective diabetes management [21]. Therefore, the achievement of glucose control should be pursued from the early stages to preserve PwD from long-term disabling complications [21, 22]. Expanding the use of CGM to all people at diabetes onset needing insulin therapy may support effective intensification of treatments valuable to reduce glucose exposure and glucose variability and lower the risk of complications and hospital admissions, which are associated with high economic burden. Of note, these goals may be achieved while minimizing the risk of hypoglycemia and improving quality of life for people with diabetes. Moreover, the analysis of glucose pattern may be used as an educational tool to demonstrate the relationship between an individual’s glucose levels, his/her medication, and other therapeutic interventions. There is evidence from observational studies that the early use (within one year from the diagnosis) of both isCGM [23] and rtCGM [24–26] results in long-term improvement in HbA_1c_ and the reduction in diabetes-related emergency-department admissions in people with type 1 diabetes. Moreover, the use of CGM at the onset of diabetes allows patients to be aware of the glycemic trends and their changes following insulin therapy. On the other hand, CGM may help physicians to monitor glycemic control and hence optimize structured therapeutic education remotely. The use of an isCGM as the first choice at diabetes onset may be preferred, as it provides a less expensive device with automatic transmission of data, does not need calibrations, and is approved for insulin dosing without SMBG. Only when symptoms of hypoglycemia do not match the glucose level detected, or glucose levels are rapidly changing, SMBG has to be considered. Moreover, isCGM with optional alarms for high and low blood glucose may be useful in patients in good metabolic control at low risk of hypoglycemia who have physical, psychological, or occupational barriers to the regular use of capillary glucose monitoring. A rtCGM should be reserved to people with a recent diabetes diagnosis and poor glucose control (i.e. high glucose variability) or disabling hypoglycemia: this should be based on the possibility to set alarms for hypoglycemia, hyperglycemia and rapid variations in glucose, increasing further the effectiveness of clinical management.

### Third scenario: type 2 diabetes

**Fig. 3:**
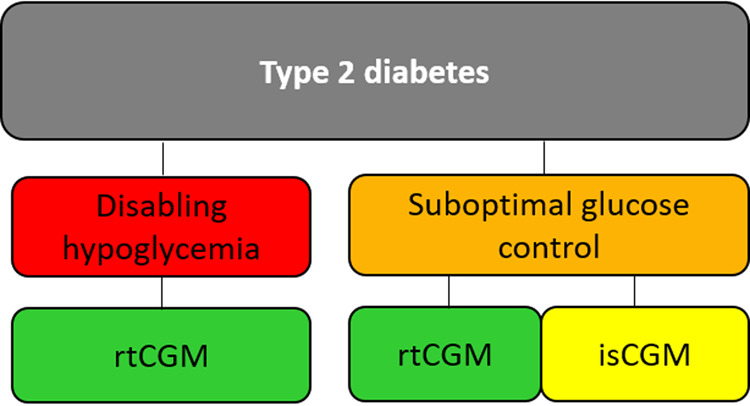
Suggested approach for the choice of CGM in type 2 diabetes

Figure 3. In patients with type 2 diabetes treated with insulin therapy in whom hypoglycemia predominates, a rtCGM with predictive alerts and alarms should be used. In type 2 PwD with suboptimal glucose control and low risk for hypoglycemia who require more data than those offered by SMBG, both isCGM and rtCGM can be suggested, depending on the individual’s circumstances, preferences, and needs.

Studies evaluating the effects of CGM in people with type 2 diabetes are limited, especially those including patients not on intensive insulin therapy [9]. There is evidence from RCTs that rtCGM reduces HbA_1c_ and increases TIR in people with type 2 diabetes treated with multiple daily injections [27] or basal insulin [9], and is more effective than SMBG in minimizing hypoglycemia for people using therapies at high hypoglycemic risk [28]. Moreover, a recent large retrospective cohort study including 41753 participants with insulin-treated diabetes, of whom 36 080 with type 2 diabetes, showed that among adults who started rtCGM, those with type 2 diabetes had greater improvements in HbA_1c_ than patients with type 1 diabetes associated with reductions in emergency department visits and hospitalizations for hypoglycemia [29]. Of note, recent practice guidelines suggest the use of rtCGM for people with type 2 diabetes who take insulin and are at risk for hypoglycemia [30]. Furthermore, the intermittent use of rtCGM in people with type 2 diabetes on oral anti-diabetic drugs, with or without basal insulin, is emerging as an effective strategy in lowering HbA_1c_ in the short-term [31, 32].

In a recent meta-analysis of RCTs, the use of isCGM was shown to significantly reduce HbA_1c_ levels of −0.74% in participants with T2D, with a lower risk of hypoglycemia compared with SMBG, and a greater effect in participants aged ≤ 65 years [33]. A significant improvement in HbA_1c_ associated with isCGM has also been found in an observational study of people with type 2 diabetes on basal-bolus therapy [34]. Evidence relative to the efficacy of isCGM in improving glycemic outcomes, including a greater TIR, a lower TBR, and a higher reduction in HbA_1c_ levels, are also emerging among non-insulin-treated people with type 2 diabetes [35]. Finally, the use of isCGM should be a valuable therapeutic opportunity in people with type 2 diabetes and fair glucose control [36].

### Fourth scenario: diabetes and pregnancy on intensive insulin therapy

**Fig. 4:**
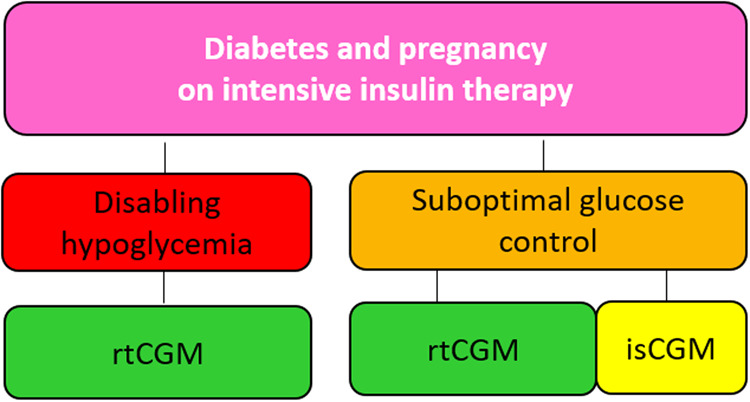
Suggested approach for the choice of CGM in diabetes and pregnancy on insulin therapy

Figure 4. In the presence of pre-gestational diabetes (both type 1 and type 2), or gestational diabetes on intensive insulin therapy and suboptimal glucose control, the preferential use of rtCGM or alternatively an isCGM should be considered in addition to SMBG in order to frequently monitor blood glucose levels and achieve glucose targets throughout pregnancy. If there is a risk of maternal hypoglycemia, a rtCGM should be preferred.

An increasing corpus of evidence demonstrates that CGM (mainly rtCGM) can improve maternal pre-natal glucose levels and neonatal outcomes in women with pre-existing diabetes or gestational diabetes [37, 38]. The critical issues traditionally associated with insulin therapy in the management of diabetes become more evident during pregnancy, based on the reduced insulin sensitivity and tighter fasting and postprandial blood glucose targets to be achieved for the prevention of maternal and neonatal complications. In addition, the International Consensus on TIR endorses ambitious glucose targets for pregnant women with pre-existing type 1 diabetes [TIR 63–140 mg/dl (> 70%), TAR > 140 mg/dl (< 25%), TBR < 63 mg/dl (< 4%), TBR < 54 mg/dl (< 1%)] or pre-existing type 2 diabetes and gestational diabetes [4]. Therefore, the use of rtCGM over isCGM should be preferred because of the accuracy of the data, the possibility of enable high and low glucose alerts and predictive alarms for hypoglycemia, allowing an intense pattern of care while minimizing the risk of maternal hypoglycemia.

### Fifth scenario: regular physical exercise

**Fig. 5:**
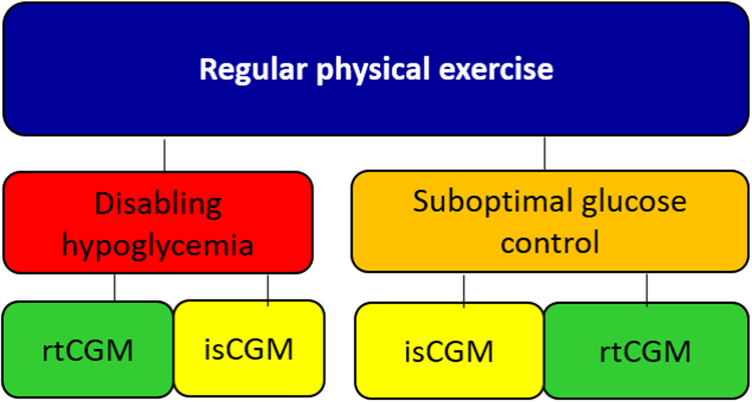
Suggested approach for the choice of CGM in regular physical activity in people with diabetes

Figure 5 Both rtCGM and isCGM should be suggested in PwD performing sporting activities, based on individual preferences and personal experiences. In case of disabling hypoglycemia in PwD performing sporting activities a rtCGM should be preferred over isCGM.

Physical activity and exercise are associated with multiple health benefits for people with diabetes [39]. CGM is emerging as an effective tool to preserve glucose homeostasis during and after exercise in PwD, thanks to the opportunity to prevent hypoglycemic events and nocturnal hypoglycemia following physical activity [40, 41]. Both rtCGM and isCGM can be effective tools to help indicate when carbohydrate intake should be started to prevent or treat hypoglycemia during exercise, as emerged in studies involving adolescents and adults with type 1 diabetes [42, 43]. Moreover, the use of rtCGM allows setting alarms to minimize the risk of hypoglycemic episodes at the onset of exercise in type 1 diabetes [40].

### Sixth scenario: frail people with diabetes

**Fig. 6:**
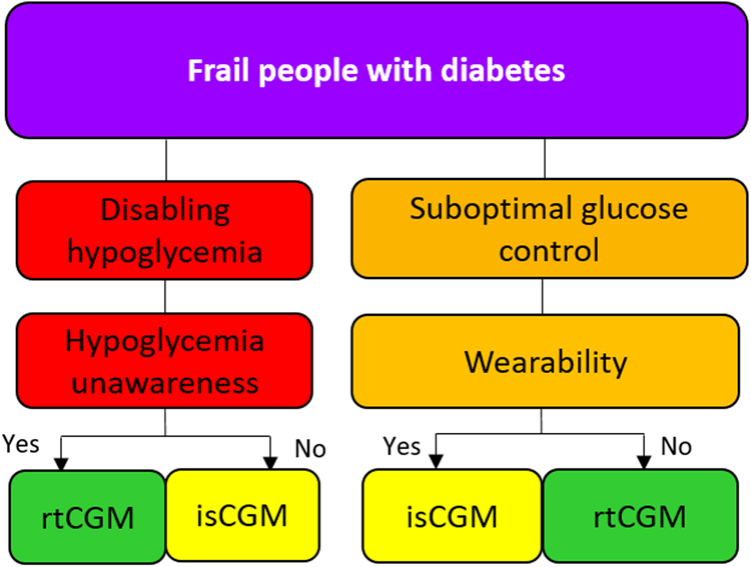
Suggested approach for the choice of CGM in frail people with diabetes

Figure 6 In frail people with insulin-treated diabetes able to use the devices (either by themselves or with a caregiver) and recurrent hypoglycemic episodes without hypoglycemia awareness, a rtCGM should be suggested in order to reduce the risk of hypoglycemic events. If the awareness of hypoglycemia is preserved, an isCGM may be considered. In the case of suboptimal glycemic control, the choice of a rtCGM or isCGM should be based on the wearability and portability of the devices and individual preferences.

Older adults with diabetes mellitus and long disease duration are at greater risk of prolonged and often unnoticed hypoglycemia, than younger adults. In order to minimize the risk of hypoglycemia, the International Consensus on TIR recommends less stringent glycemic targets in this subgroup [TIR > 50%, TBR (< 70 mg/dl) < 1%, TAR level 1 (> 180 mg/dl) < 50%, TAR level 2 (> 250 mg/dl) < 1] [4]. Evidence from Wireless Innovation in Seniors with Diabetes Mellitus (WISDM) and observational data from DCCT/EDIC study report the effectiveness of CGM in reducing the risk of hypoglycemia up-to one year [15, 18], hyperglycemic excursions and in reaching desired HbA_1c_ levels [44] in older people with type 1 diabetes. The benefits associated with CGM, in terms of improved HbA_1c_ and reduced glycemic variability, are also emerging in studies involving older people with type 2 diabetes using insulin [28, 45]. Particularly, the use of a rtCGM with alerts or predictive alarms by the user and/or the caregiver represents one of the most useful therapeutic tools to avoid hypoglycemia or mitigate its severity in this high-risk population of PwD. Emerging evidence reports the effectiveness of CGM in lowering mean glucose levels during and after hemodialysis in people with type 2 diabetes and stage 3–5 chronic kidney disease (CKD) [46, 47]. This is relevant, as CGM can reveal the daily glucose trends for PwD and CKD on hemodialysis and help them manage daily diet and physical activity.

## Discussion

Information collected from CGM is of paramount importance to guide the management of PwD experiencing recurrent hypoglycemia or glucose variability, over the control of HbA_1__c_. CGM allows patients to recognize patterns of blood glucose changes and, consequently, to adjust diet, exercise and insulin dosing accordingly. CGM contributes to improve the lives and health of PwD. On the basis of the growing evidence, its use is currently recognized as the standard of care for people with type 1 diabetes and for a subset of those with insulin-requiring type 2 diabetes [1, 2, 48].

Since the technology of continuous glucose sensors is rapidly evolving, the suggested approach for the responsible use of CGM in people with diabetes is no “one-size-fits-all” [2]. Both rtCGM and isCGM have their own features, abilities, and limitations, that must be considered when selecting the system that meets the personal and clinical needs of people living with diabetes. The present algorithm may help to select the best CGM device based on patients’ clinical characteristics, needs and clinical context, offering a further opportunity of a “tailored” therapy for people with insulin-treated diabetes. The proposed flow-charts have been elaborated on the basis of both current scientific evidence, coming mainly from clinical trials, and principles of good clinical practice, as inspired from daily experience in dealing with PwD.

rtCGM remains the preferred choice over isCGM for monitoring and detection of hypoglycemia, based on the possibility to set predictive alarms/alerts [5]. Interestingly, in the few studies comparing rtCGM with isCGM, an improvement of both TBR and TIR have been associated to rtCGM [49–51]. On the other hand, isCGM should be considered as a valuable option over rtCGM at the onset of diabetes, in patients treated with glucose-lowering therapy not associated with hypoglycemic risk or in people with suboptimal glucose control and low risk for hypoglycemia. In these situations, isCGM may provide more data than those obtained by SMBG with an higher ease of use and greater acceptability [5].

Some critical issues still remain and should be taken into account. Most RCTs reported the effects of rtCGM in PwD, whereas RCT data for isCGM are more limited. On the other hand, a higher corpus of evidence from observational/retrospective studies and analyses of registry and population data refer to the use of isCGM in adults with diabetes [34, 52–54], whereas real-world evidence for rtCGM use is still restricted to few studies [29, 55].

Future CGM devices may use nanoparticle sensors that demonstrated excellent glucose response in the physiological range and are a promising tool for real-time glucose tracking [56, 57]. In the meantime, the proposed algorithm may represent a good companion for clinicians who desire to optimize the use of CGM in daily clinical practice.

### Supplementary information

liveCGM_supplemental material
