# Correction: An updated algorithm for an effective choice of continuous glucose monitoring for people with insulin-treated diabetes

**DOI:** 10.1007/s12020-023-03635-w

**Published:** 2024-01-31

**Authors:** Maria Ida Maiorino, Raffaella Buzzetti, Concetta Irace, Luigi Laviola, Nicola Napoli, Dario Pitocco, Katherine Esposito, on behalf of LIVE CGM working group

**Affiliations:** 1 Unit of Endocrinology and Metabolic Diseases, University Hospital Luigi Vanvitelli, Piazza Miraglia 2, 80138 Naples, Italy; 2 Department of Advanced Medical and Surgical Sciences, University of Campania Luigi Vanvitelli, Piazza Miraglia 2, 80138 Naples, Italy; 3 Department of Experimental Medicine, “Sapienza” University of Rome, Viale Regina Elena 324, 00161 Rome, Italy; 4 Department of Health Science, University Magna Graecia, Viale Europa, 88100 Catanzaro, Italy; 5 Department of Precision and Regenerative Medicine and Ionian Area, Section of Internal Medicine, Endocrinology, Andrology and Metabolic Diseases, University of Bari Aldo Moro, Piazza Giulio Cesare 11, 70124 Bari, Italy; 6 Fondazione Policlinico Universitario Campus Bio-Medico, Research Unit of Endocrinology and Diabetes, Department of Medicine and Surgery, Università Campus Bio-Medico of Rome, Via Alvaro del Portillo 21, 00128 Rome, Italy; 7 Diabetes Care Unit, Department of Translational Medicine and Surgery, Università Cattolica del Sacro Cuore, Fondazione Policlinico Universitario Agostino Gemelli IRCCS, Largo Francesco Vito 1, 00168 Rome, Italy

Correction to: Endocrine (2023) 82:215–225

10.1007/s12020-023-03473-w

In this article, the mean absolute relative difference (MARD) for the rtCGM/Glunovo i3/Flash device in Table1, column MARD, was initially reported as 9.3%. The value has been rectified to 10.3% in this erratum.

**Table S1**. Characteristics of currently available CGM devices

**Table:** 

CGM type/sensor	Reading period/sensor lifetime/transmitter lifetime	Calibrations (*n*/day)	Smartphone compatibility/ CSII integrated (SAP/AP)	MARD	Insertion site	% 15/15 mg/dl overall^e^	% 20/20 mg/dl overall^f^	Data management	Adjunctive use/non adjunctive use	Use in pregnancy^i^	Intended populations
rtCGM/Dexcom G6	10 days/10 days/ 3 months	0	Yes/Yes	9%	Abdomen, arm, upper gluteus^d^	83.3%	93.9%	Clarity/Diasend/ Glooko	Non adjunctive use	Indicated	2 years and older
rtCGM/Dexcom G7	10 days/10 days^b^ (+12 h tolerance)	0	Yes/Yes	8.2%	Abdomen, arm, upper gluteus^d^	89.6%^g^	95.3%^g^	Clarity/ Glooko	Non adjunctive use	Indicated	2 years and older
rtCGM/TouchCare A7+	10 days /10 days/ 12 months	1	Yes/Yes	9%	Abdomen, upper gluteus	NA	NA	EasyView Pro	Non adjunctive use	Not specified	2 years and older
rtCGM/Eversense E3^a^	180 days/180 days/12 months	2/1^c^	Yes/No	8.5%	Upper arm	85.6%	92.9%	Eversense DMS	Non adjunctive use	Not indicated	18 years and older
rtCGM/GlucoMen Day	14 days/14 days/ 5 years	1	Yes/No	9.6%	Abdomen	NA	NA	Glucolog/Diasend/ Glooko	Non adjunctive use	Indicated	6 years and older
rtCGM/Glunovo i3/Flash	14 days/14 days/ 3 years	0	Yes/No	10.3%	Abdomen	79.31%	89.71%	IrisHealthCare	Adjunctive use	Indicated	2 years and older
rtCGM/Guardian sensor 3	7 days/7 days/ 12 months	2	No/Yes	9.1%	Abdomen, arm	78.8%	88.2%	Carelink	Adjunctive use^h^	Not specified	2 years and older
rtCGM/Guardian sensor 4	7 days/7 days/ 12 months	0	No/Yes	10.6%	Abdomen, arm, upper gluteus^d^	NA	NA	Carelink	Non adjunctive use	Not specified	2 years and older
iCGM/Freestyle libre	14 days/14 days^b^	0	Yes/No	9.2%	Arm	NA	NA	Libreview	Non adjunctive use	Indicated	4 and older
iCGM/Freestyle libre 2	14 days/14 days^b^	0	Yes/No	9.2%	Arm	86.3%	93.2%	Libreview	Non adjunctive use	Indicated	4 and older
rtCGM/Freestyle libre 3	14 days/14 days^b^	0	Yes/No	7.9%	Arm	89.1%	94.7%	Libreview	Non adjunctive use	Indicated	4 and older

*AP* artificial pancreas, *CGM* continuous glucose monitoring, *CSII* continuous subcutaneous insulin infusion, *iCGM* intermittently scanned glucose monitoring, *MARD* mean absolute relative difference, *rtCGM* real-time continuous glucose monitoring, *SAP* sensor augmented pump.

^a^Implantable sensor.

^b^The sensor and the transmitter work as one unit.

^c^Eversense E3 requires 2 calibration/day for the first 21 days of use and after 1 calibration/day.

^d^Upper gluteus refers to pediatric use between 2 and 17 years of age.

^e^Overall agreement rate between paired sensor–reference values within ±15%/15%.

^f^Overall agreement rate between paired sensor–reference values within ±20%/20%.

^g^Overall agreement rate for arm-placed sensors.

^h^Approved for non adjunctive use only in conjunction with MiniMed 780G.

^i^Use in pregnancy according to the technical sheet.

The original article has been corrected.

