# Supplementary material for: An updated algorithm for an effective choice of continuous glucose monitoring for people with insulin-treated diabetes
Source: Endocrine. 2023 Sep 7;82(2):215–25. doi: 10.1007/s12020-023-03473-w (PMC10543826; doi:10.1007/s12020-023-03473-w)
Supplement: Supplementary file 1 — liveCGM_supplemental material [file 12020_2023_3473_MOESM1_ESM.docx]

An updated a**l**gor**i**thm for an effecti**ve** choice of **c**ontinuous **g**lucose **m**onitoring for people with insulin-treated diabetes

*Supplementary material*

*Composition of LIVE CGM Working group……………………………………………………………………………pag. 2*

*Composition of LIVE CGM Working group*

Giuseppe Bellastella, Unit of Endocrinology and Metabolic Diseases, Department of Advanced Medical and Surgical Sciences, University of Campania Luigi Vanvitelli, Naples, Italy; [giuseppe.bellastella@unicampania.it](mailto:giuseppe.bellastella@unicampania.it)

Paola Caruso, Unit of Endocrinology and Metabolic Diseases, Department of Advanced Medical and Surgical Sciences, University of Campania Luigi Vanvitelli, Naples, Italy; [paola.caruso@unicampania.it](mailto:paola.caruso@unicampania.it)

Filomena Castaldo, Unit of Endocrinology and Metabolic Diseases, University Hospital of Campania Luigi Vanvitelli, Naples, Italy; [dot.castaldof86@gmail.com](mailto:dot.castaldof86@gmail.com)

Sergio Di Molfetta, Department of Precision and Regenerative Medicine and Ionian Area, Section of Internal Medicine, Endocrinology, Andrology and Metabolic Diseases, University of Bari Aldo Moro, Bari, Italy, [s.dimolfetta@libero.it](mailto:s.dimolfetta@libero.it)

Miriam Longo, Unit of Endocrinology and Metabolic Diseases, Department of Advanced Medical and Surgical Sciences, University of Campania Luigi Vanvitelli, Naples, Italy; [miriam.longo@unicampania.it](mailto:miriam.longo@unicampania.it)

Carmen Mignogna, Department of Experimental Medicine, "Sapienza" University of Rome, Rome, Italy, [carmen.mignogna@uniroma1.it](mailto:carmen.mignogna@uniroma1.it)

Martina Parise, Department of Health Science, University Magna Graecia, Catanzaro, Italy

[parise.martina@gmail.com](mailto:parise.martina@gmail.com)

Michela Petrizzo, Unit of Endocrinology and Metabolic Diseases, University Hospital of Campania Luigi Vanvitelli, Naples, Italy; [michela.petrizzo@unicampania.it](mailto:michela.petrizzo@unicampania.it)

Silvia Pieralice, Fondazione Policlinico Campus Biomedico, Rome, Italy,

Alessandro Rizzi, Diabetes Care Unit Fondazione Policlinico Universiatrio IRCCS, [alessandro.rizzi@unicatt.it](mailto:alessandro.rizzi@unicatt.it)

Lorenzo Scappaticcio, Department of Advanced Medical and Surgical Sciences, University of Campania Luigi Vanvitelli, Naples, Italy; [lorenzo.scappaticcio@unicampania.it](mailto:lorenzo.scappaticcio@unicampania.it)

Linda Tartaglione, Diabetes Care Unit Fondazione Policlinico Universiatrio IRCCS, [linda.tartaglione@policlinicogemelli.it](mailto:linda.tartaglione@policlinicogemelli.it)
